# Short-time-window Patlak imaging using a population-based arterial input function and optimized Bayesian penalized likelihood reconstruction: a feasibility study

**DOI:** 10.1186/s13550-022-00933-8

**Published:** 2022-09-08

**Authors:** Takato Tanaka, Masatoyo Nakajo, Hirofumi Kawakami, Eriko Motomura, Tomofumi Fujisaka, Satoko Ojima, Yasumasa Saigo, Takashi Yoshiura

**Affiliations:** 1 Department of Radiation Technology, Kagoshima University Hospital, 8-35-1 Sakuragaoka, Kagoshima, 890-8544 Japan; 2 Department of Radiology, Graduate School of Medical and Dental Sciences, Kagoshima University, 8-35-1 Sakuragaoka, Kagoshima, 890-8544 Japan; 3 Academic Department, GE Healthcare Japan, 4-7-127 Asahigaoka-Hinoshi, Tokyo, 191-8503 Japan; 4 Department of Cardiovascular Medicine and Hypertension, Graduate School of Medical and Dental Sciences, Kagoshima University, 8-35-1 Sakuragaoka, Kagoshima, 890-8544 Japan

**Keywords:** Dynamic ^18^F-FDG-PET/CT, Patlak Ki image, Population-based input function, Individual patient-based input function, Bayesian penalized likelihood reconstruction

## Abstract

**Background:**

To explore the feasibility of short-time-window Ki imaging using a population-based arterial input function (IF) and optimized Bayesian penalized likelihood (BPL) reconstruction as a practical alternative to long-time-window Ki imaging with an individual patient-based IF. Myocardial Ki images were generated from 73 dynamic ^18^F-FDG-PET/CT scans of 30 patients with cardiac sarcoidosis. For each dynamic scan, the Ki images were obtained using the IF from each individual patient and a long time window (10–60 min). In addition, Ki images were obtained using the normalized averaged population-based IF and BPL algorithms with different beta values (350, 700, and 1000) with a short time window (40–60 min). The visual quality of each image was visually rated using a 4-point scale (0, not visible; 1, poor; 2, moderate; and 3, good), and the Ki parameters (Ki-max, Ki-mean, Ki-volume) of positive myocardial lesions were measured independently by two readers. Wilcoxon’s rank sum test, McNemar’s test, or linear regression analysis were performed to assess the differences or relationships between two quantitative variables.

**Results:**

Both readers similarly rated 51 scans as positive (scores = 1–3) and 22 scans as negative (score = 0) for all four Ki images. Among the three types of population-based IF Ki images, the proportion of images with scores of 3 was highest with a beta of 1000 (78.4 and 72.5%, respectively) and lowest with a beta of 350 (33.3 and 23.5%) for both readers (all *p* < 0.001). The coefficients of determination between the Ki parameters obtained with the individual patient-based IF and those obtained with the population-based IF were highest with a beta of 1000 for both readers (Ki-max, 0.91 and 0.92, respectively; Ki-mean, 0.91 and 0.92, respectively; Ki-volume, 0.75 and 0.60, respectively; and all *p* < 0.001).

**Conclusions:**

Short-time-window Ki images with a population-based IF reconstructed using the BPL algorithm and a high beta value were closely correlated with long-time-window Ki images generated with an individual patient-based IF. Short-time-window Ki images using a population-based IF and BPL reconstruction might represent practical alternatives to long-time-window Ki images generated using an individual patient-based IF.

**Supplementary Information:**

The online version contains supplementary material available at 10.1186/s13550-022-00933-8.

## Introduction

Glucose metabolic activity is reflected by ^18^F-fluorodeoxyglucose (^18^F-FDG) uptake during positron emission tomography (PET)/computed tomography (CT) in both oncological and inflammatory disorders [1, 2]. The two most widely used quantitative indices of ^18^F-FDG metabolism are the standardized uptake value (SUV) [3, 4] and Ki, which represents the rate of ^18^F-FDG uptake and is a quantitative index of ^18^F-FDG metabolism measured using the Patlak slope [5, 6]. SUV is a simple semiquantitative index that is calculated by measuring the activity in the lesion during a short-duration static scan acquired late (typically 60 min) after injection and then normalized for the injected dose and either patient weight or lean body mass [7, 8]. SUV measures the total activity in the lesion, and it includes both ^18^F-FDG and non-metabolized ^18^F-FDG (unphosphorylated ^18^F-FDG) in the blood, intercellular spaces, and/or cells [9].

Conversely, Patlak analysis separates these two components, and the Patlak slope is determined using only metabolized ^18^F-FDG [4]. Thus, measurements of Ki might contribute to assessments of disease activity, and dynamic PET has been used in oncological or inflammatory disorders to characterize the kinetic ^18^F-FDG model [10, 11]. However, Ki images are considered inconvenient for patients because they require dynamic imaging to obtain an arterial input function (IF) with the construction of a lesion time–activity curve [5, 6] and they exhibit high image noise [12].

van Sluis et al. [13] explored the effects of various simulated population-averaged IF on the accuracy of Patlak analysis based on dynamic whole-body PET acquisition from 30 to 60 min and reported that scaling of a population-averaged IF to IF values seen in whole-body dynamic imaging from 30 to 60 min post-injection can provide accurate Ki estimates. However, the robust or feasible population-averaged IFs models for proceeding the Patlak analysis in clinical routine practice have not yet been established. Thus, the use of a population-averaged IF could obviate the need for the first dynamic scan and shorten the scan time for Patlak analysis [13, 14], making dynamic Patlak imaging clinically feasible.

The Bayesian penalized likelihood (BPL) reconstruction algorithm is a new PET reconstruction method that can be used to improve clinical image quality and quantification [15]. One of the main characteristics of the BPL algorithm is the suppression of noise inside the iterative reconstructions, which allows an increased number of iterations without increasing the noise, and therefore, full convergence can be achieved [16]. Thus, applying a population-based IF and the BPL reconstruction algorithm to Ki images could potentially eliminate the aforementioned problems, lessen patient inconvenience, and reduce image noise. However, to our knowledge, no study has examined whether the optimized BPL reconstruction algorithm could improve the image quality in Ki images.

Therefore, this study explored the feasibility of short-time-window Ki imaging using a population-based IF and optimized BPL algorithm as a practical alternative to long-time-window Ki imaging using an individual patient-based IF.

## Materials and methods

### Study design and patient selection

This retrospective study was approved by the ethics committee on epidemiological studies of our institution, which waived the requirement for informed consent. This study enrolled consecutive 30 patients who underwent dynamic ^18^F-FDG-PET/CT to assess the disease activity of cardiac sarcoidosis (CS) from April 2019 to January 2021. These patients underwent a combined 75 ^18^F-FDG-PET/CT scans. Patients with incomplete dynamic scans were excluded.

In a previous study [11], the usefulness of Patlak Ki images derived from an individual patient-based IF for evaluating the risk of clinical events was examined in 21 patients with CS who underwent 30 ^18^F-FDG-PET/CT scans, and patients were enrolled between April 2019 and January 2020. These 30 ^18^F-FDG-PET/CT scans were included in the 75 ^18^F-FDG-PET/CT scans, because analyses of the differences or relationships in Ki images between the individual patient-based IF with a long time window and the population-based IF with a short time window were not examined in these 30 ^18^F-FDG-PET/CT scans in the previous study [11].

Two ^18^F-FDG-PET/CT scans were excluded because of incomplete dynamic ^18^F-FDG-PET/CT scans. Finally, 30 patients (22 women and 8 men; mean age, 62 ± 11 years; age range, 39 − 78 years) with 73 ^18^F-FDG-PET/CT scans were enrolled. The number of scans was one, two, three, four, and five in 10, 4, 10, 5, and 1 patients, respectively.

### Imaging protocols

All patients were instructed to fast for ≥ 18 h before PET/CT, which resulted in a mean plasma glucose level of 106 mg/dl (range, 56–167 mg/dl) immediately before the ^18^F-FDG intravenous injection.

All ^18^F-FDG PET/CT examinations were performed on a Discovery MI PET/CT (GE Healthcare, Milwaukee, WI, USA). First, low-dose CT covering the entire heart was performed (slice thickness, 3.75 mm; pitch, 1.375 mm; 120 keV; auto mA (40–100 mA depending on patient body mass); reconstructed matrix size, 512 × 512) with the transaxial and craniocaudal fields of view (FOVs) of 70 and 20 cm, respectively, which were used for attenuation correction of the PET images. Thereafter, ^18^F-FDG [230 ± 26 MBq (range, 162–286 MBq)] was injected, and dynamic list-mode PET data (single-bed) covering the aforementioned craniocaudal FOV were acquired with the following PET frames. The acquisition began at the time of injection, with scan times of 10 s/frame for the first 2 min, 3 min/frame for the next frame, and 5 min/frame thereafter for a total of 60 min. The motion corrections including body motion and respiratory motion were not performed for the dynamic PET data. The PET transaxial spatial resolution was 3.9 mm full-width half-maximum in-plane. The registration of CT and reconstructed dynamic PET image was verified using the Attenuation Correction Quality Control (ACQC) application (GE Healthcare) on the PET/CT scanner.

### Calculation of Ki

To determine the ^18^F-FDG kinetic parameters within each lesion, a linear approximation of the mathematical representation of the standard two-compartmental model with irreversible trapping was used according to Patlak analysis [5].

From C_i_ (*tk*), the ^18^F-FDG activity concentration in the lesion (Bq mL_tissue_^−1^) at a given time *tk* after injection, the analytical solution of the two-compartment model is given as:

$$C_{i} \left( {tk} \right) = {\text{Ki}}\int\limits_{0}^{tk} {C_{{\text{p}}} \left( t \right){\text{d}}t + V_{{\text{p}}} C_{{\text{p}}} \left( {tk} \right)}$$

where *C*_p_(*tk*) represents the ^18^F-FDG activity concentration in blood plasma at time *tk* (Bq mL_blood_^−1^) and *V*_p_ is the total blood distribution volume (i.e., the unmetabolized fraction of ^18^F-FDG in blood and interstitial volume).

The compartmental transfer rates, namely *K*1 (from blood to cell), *k*2 (from cell to blood), and *k*3 (from ^18^F-FDG to ^18^F-FDG-6-phosphate), were used to calculate Ki, the net influx rate, as follows: Ki = (*K*1 × *k*3)/(*k*2 + *k*3). The transfer rate *k*4 from ^18^F-FDG-6-phosphate to ^18^F-FDG is negligible because Patlak analysis assumes unidirectional uptake of ^18^F-FDG (*k*4 = 0). The Ki unit is ml/g/min.

### Generation of Ki images with the individual patient-based IF as the reference images

The individual patient IF was determined by blood time–activity curves derived from PET [image-derived input functions (IDIFs)] as described previously [11], and the following region of interest (ROI) was set to determine the IF by one radiologic technician. The investigator was aware of the study purpose but blinded to clinical information. A 15-mm-diameter spherical ROI was manually drawn in the center of the ascending aorta on the registered image to reduce contaminants such as atherosclerotic plaques or smooth muscles in the arterial wall. Patlak analysis was performed over the period from 10 to 60 min after injection during steady state. The data were reconstructed using time of flight (TOF) with the BPL reconstruction algorithm including point spread function (PSF) modeling, a beta value of 700, and transaxial FOV of 50 cm as the individual patient-based Ki image. The matrix size was 128 × 128, and the voxel size was 3.91 × 3.91 × 2.78 mm^3^. The Ki images generated using the individual patient-based IF (hereafter individual patient-based IF Ki images) were used as the reference images for evaluated Ki images generated using the population-based IF (population-based IF Ki images).

### Generation of population-based IF Ki images

The normalized average of the arterial IF was used as the population-based IF. The population-based IF was generated from the individual IFs of the first 12 patients during the inclusion period acquired using the aforementioned protocol by the following method; the correlations between IDIF [$\int\limits_{0}^{50} {C_{{\text{p}}} \left( t \right){\text{d}}t}$ (integral of plasma activity *C*_p_ from time 0 to 50)] and plasma activity of aorta at the uptake time of 50 min (Ao-50) were analyzed by the linear regression analysis among these 12 patients. Thereafter, the individual population-based IF was determined using the acquired regression equation (*Y* = 113.76 + 97.16*x*; *r* = 0.98, *p* < 0.001) (Additional file 1: Fig. S1).

To generate the population-based IF Ki images, Patlak analysis was performed over the period from 40 to 60 min after ^18^F-FDG injection with the following reconstruction method; The dynamic PET data from 40 to 60 min post-injection were reconstructed into 3 frames (6 min, 7 min and 7 min) using the same matrix and FOV used for individual patient-based IF Ki images. The images were reconstructed using TOF with the BPL algorithms including PSF modeling with three different penalization factors (beta values of 350, 700, and 1000). Thus, three population-based IF Ki images were created using these beta values for each study (Ki-350, Ki-700, and Ki-1000).

### Image analysis

**Fig. 1:**
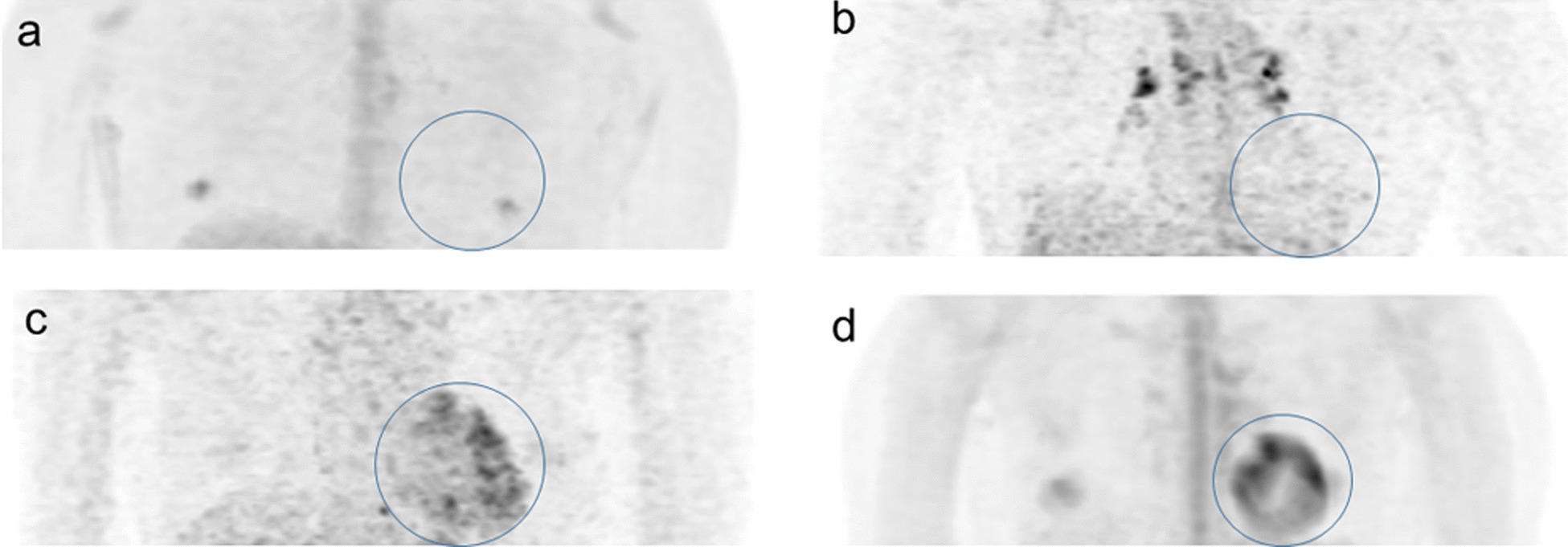
Myocardial visual quality on Ki images using a 4-point scale: 0 (**a**, circle), myocardium not visible; 1 (**b**, circle), poor lesion conspicuity; 2 (**c**, circle), moderate conspicuity; and 3 (**d**, circle), good conspicuity. The visible focal spot in the circle of panel (**a**) indicates the nipple

One nuclear medicine technician and one nuclear medicine radiologist who were aware of the study purpose but blinded to clinical information interpreted the Ki images independently. Four different Ki images including one individual patient-based and three population-based IF Ki images were read simultaneously for each study. The researchers assessed myocardial visual quality for each Ki image using a four-point scale as follows: 0, myocardium not visible; 1, poor lesion conspicuity, the degree of myocardial ^18^F-FDG uptake is above background but is difficult to distinguish from background noise; 2, moderate conspicuity, the degree of myocardial ^18^F-FDG uptake is above background and distinguishable from background noise; and 3, good conspicuity, the degree of myocardial ^18^F-FDG uptake is above background and distinguishable from noise and the lesion circumference is definable [17] (Fig. 1). Scores of 0 and 1–3 were assigned as negative and positive, respectively.

For the images interpreted as positive by the two independent observers, the following quantitative parameters were obtained: {maximum Ki (Ki-max), mean Ki, [(Ki-mean), and volume of Ki (Ki-volume)]}. Each observer set the volumes of interest (VOIs) for the four Ki images for each study independently. They manually placed the VOIs on a suitable reference fused axial image and then defined the craniocaudal and mediolateral extent encompassing the entire positive myocardial lesion, excluding any avid extracardiac structures, to obtain Ki-max. They next set a 40% threshold of Ki-max to automatically delineate a VOI equal to or greater than the 40% threshold of Ki-max to calculate Ki-mean and Ki-volume, respectively. In the quantitative analysis of Ki images, the same method for VOI setting was applied for each Ki image. Workstations (Xeleris or Advantage Windows Workstation 4.5; GE Healthcare) automatically calculated Ki-max, Ki-mean, and Ki-volume.

### Statistical analysis

The inter-observer agreement of the visual rating was evaluated using *κ* statistics analysis, and *κ* was interpreted as follows: less than 0.20, slight agreement; 0.21–0.40, fair agreement; 0.41–0.60, moderate agreement; 0.61–0.80, substantial agreement; and 0.81 or greater, almost perfect agreement [18]. Linear regression analysis was used to assess the relationship between two quantitative variables. The Wilcoxon rank sum test was used to assess the difference between two quantitative variables. The McNemar test was used to examine differences in the rating of visual scores among the four Ki images.

Data were presented as mean and standard deviation. *p* < 0.05 was considered indicative of statistical significance, and all *p* values were two-tailed. MedCalc Statistical Software (MedCalc Software Ltd., Acacialaan 22, 8400 Ostend, Belgium) was used for the statistical analyses.

## Results

### Visual quality of each Ki image

The visual scores for the Ki images are summarized in Table 1.

**Table 1:** Visual findings among the Ki images

	Reader 1				Reader 2			
	Visual score				Visual score			
Ki image	0	1	2	3	0	1	2	3
Individual patient-based Ki image	22	0	0	51	22	0	0	51
*Population-based Ki image*								
Ki-350 images	22	7	27	17	22	14	25	12
Ki-700 images	22	0	14	37	22	0	15	36
Ki-1000 images	22	0	11	40	22	0	14	37

For the individual patient-based IF Ki images (reference images), both readers 1 and 2 awarded a score of 3 to 51 scans and a score of 0 to 22 scans. Inter-observer agreement was perfect for the reference images between the two readers with *κ* of 1.00 [95% confidence interval (CI) = 1.00–1.00].

For the Ki-350 images, reader 1 gave scores of 3, 2, 1, and 0 to 17, 27, 7, and 22 scans, respectively, whereas reader 2 awarded scores of 3, 2, 1, and 0 to 12, 25, 14, and 22 scans, respectively. The inter-observer agreement was substantial for the Ki-350 image (*κ* = 0.80, 95% CI = 0.71–0.89). Both readers rated 51 scans as positive and 22 scans as negative, and this positive and negative rating was the same as that of the individual patient-based IF Ki images.

For the Ki-700 images, reader 1 gave scores of 3, 2, and 0 to 37, 14, and 22 scans, respectively, whereas reader 2 gave scores of 3, 2 and 0 to 36, 15, and 22 scans, respectively. The inter-observer agreement was almost perfect (*κ* = 0.87, 95% CI = 0.79–0.95). Both readers rated 51 scans as positive and 22 scans as negative, and this positive and negative rating was the same as that of the individual patient-based IF Ki images.

For the Ki-1000 images, reader 1 gave scores of 3, 2, and 0 to 40, 11, and 22 scans, respectively, whereas reader 2 gave scores of 3, 2, and 0 to 37, 14, and 22 scans, respectively. The inter-observer agreement was almost perfect (*κ* = 0.87, 95% CI = 0.78–0.95). Thus, both readers rated 51 scans as positive and 22 scans as negative, and this positive and negative rating was the same as that of the individual patient-based IF Ki images.

Among the population-based IF Ki images (Ki-350, Ki-700, and Ki-1000 images), the proportion of scans with a score of 3 was highest for Ki-1000 images (78.4 [40/51] and 72.5% [37/51] for readers 1 and 2, respectively) and lowest for Ki-350 images (33.3 [17/51] and 23.5% [12/51], respectively). There were significant differences in the proportion of scans with scores of three between the reference images and all three population-based Ki images for both readers (all *p* < 0.05). Among the population-based Ki images, there were significant differences in the proportion of scans with a score of 3 between Ki-350 and Ki-1000 images and between Ki-350 and Ki-700 images for both readers (all *p* < 0.001). No differences in the number of scans with a score of 3 were observed between Ki-700 and Ki-1000 images for either reader (reader 1, *p* = 0.38; reader 2, *p* = 1.00).

**Fig. 2:**
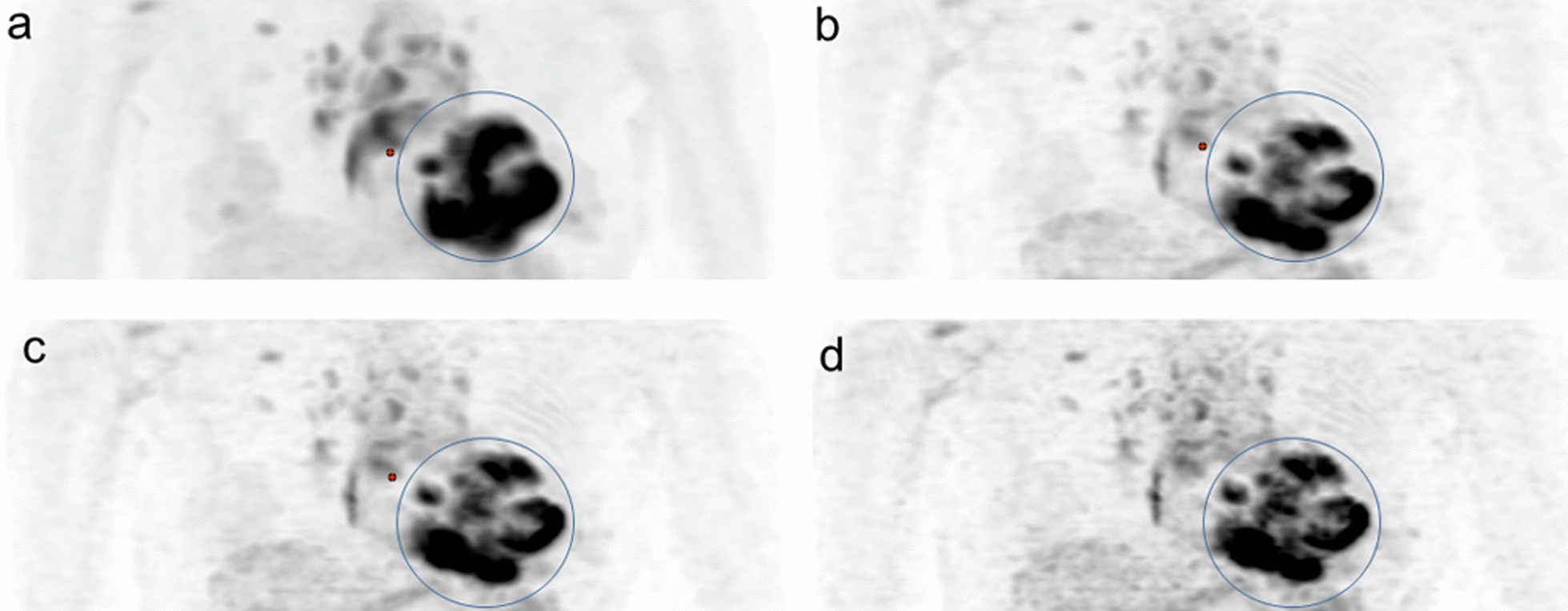
A 72-year-old female patient with cardiac sarcoidosis before treatment. The individual patient-based Ki image (**a**) and three population-based Ki images [Ki-1000 image (**b**), Ki-700 image (**c**), and Ki-350 image (**d**)] all had a visual score of 3 in the myocardium (circles)

**Fig. 3:**
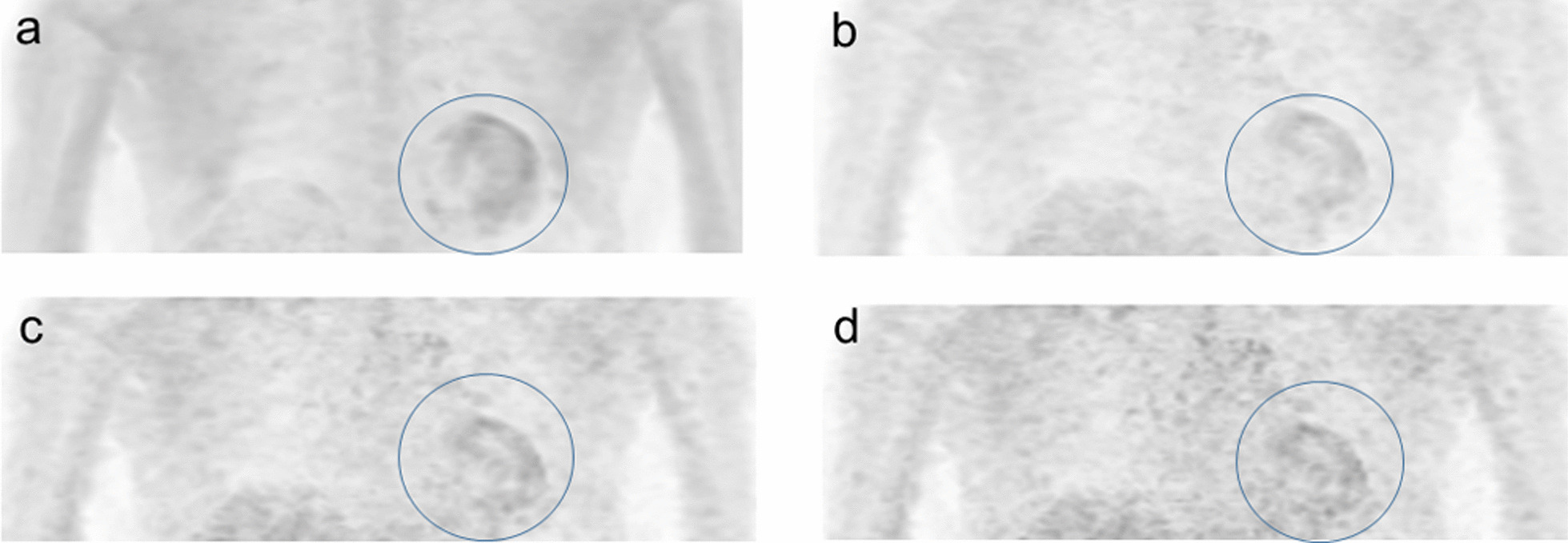
A 51-year-old male patient with cardiac sarcoidosis under steroid treatment. The individual patient-based Ki image (**a**) had a visual score of 3 in the myocardium (circle). Both the Ki-1000 (**b**) and Ki-700 images (**c**) had a visual score of 2 (circles), whereas the Ki-350 image (**d**) had a visual score of 1 (circle)

The Ki images of the representative scans are presented in Figs. 2 and 3.

### Correlations between quantitative parameters

The three Ki parameters (Ki-max, Ki-mean, and Ki-volume) of each Ki image and the correlation (*r*) and coefficient of determination (*r*^2^) for Ki parameter values between individual patient- and population-based IF Ki images for the 51 positive scans are summarized in Table 2.

**Table 2:** Each Ki parameter value and correlations of Ki parameter values in individual patient-based and population-based Ki images of 51 positive scans

	Reader 1	Reader 2
Individual patient-based Ki image	Mean ± SD (range)	Mean ± SD (range)
Ki-max (× 10^−3^)^a^	149.5 ± 116.3 (47.4–545.4)	153.6 ± 120.3 (47.4–552.0)
Ki-mean (× 10^−3^)^a^	83.8 ± 70.1 (27.0–333.0)	85.0 ± 71.4 (25.8–326.4)
Ki-volume^b^	56.0 ± 29.0 (2.4–118.0)	59.7 ± 28.5 (2.4–115.0)

Population-based Ki image	Mean ± SD (range)	Linear regression^c^			Mean ± SD (range)	Linear regression^c^		
		*r*	*r*^2^	*p*		*r*	*r*^2^	*p*
*Ki-350 images*								
Ki-max (× 10^−3^)^a^	245.5 ± 149.9 (88.2–701.4)	0.93	0.86	< 0.001	278.6 ± 157.4 (88.2–701.4)	0.90	0.80	< 0.001
Ki-mean (× 10^−3^)^a^	128.9 ± 85.0 (46.2–403.2)	0.91	0.83	< 0.001	132.2 ± 82.6 (46.2–404.4)	0.89	0.79	< 0.001
Ki-volume^b^	31.4 ± 19.6 (1.1–84.5)	0.84	0.70	< 0.001	30.9 ± 18.8 (1.1–82.8)	0.75	0.57	< 0.001
*Ki-700 images*								
Ki-max (× 10^−3^)^a^	228.1 ± 142.6 (76.2–671.4)	0.94	0.89	< 0.001	242.2 ± 138.9 (82.2–672.0)	0.94	0.88	< 0.001
Ki-mean (× 10^−3^)^a^	121.4 ± 90.0 (39.6–392.4)	0.94	0.88	< 0.001	124.4 ± 80.8 (40.8–394.8)	0.95	0.89	< 0.001
Ki-volume^b^	30.0 ± 21.3 (1.4–94.3)	0.82	0.67	< 0.001	38.7 ± 22.4 (1.4–91.6)	0.73	0.54	< 0.001
*Ki-1000 images*								
Ki-max (× 10^−3^)^a^	206.7 ± 140.0 (64.8–654.6)	0.95	0.91	< 0.001	219.5 ± 136.8 (64.8–654.0)	0.96	0.92	< 0.001
Ki-mean (× 10^−3^)^a^	110.8 ± 79.7 (33.6–384.6)	0.95	0.91	< 0.001	113.7 ± 79.8 (33.6–387.0)	0.96	0.92	< 0.001
Ki-volume^b^	41.8 ± 23.3 (1.7–96.0)	0.87	0.75	< 0.001	41.3 ± 22.6 (1.8–95.4)	0.78	0.60	< 0.001

SD, Standard deviation; Ki-max, Maximum Ki; Ki-mean, Mean Ki; Ki-volume, Volume of Ki; *r*, Correlation coefficient; and *r*^2^, Coefficient of determination

^a^The unit of Ki-max and Ki-mean is ml/g/min

^b^The unit of Ki-volume is cm^3^

^c^The linear regression between the patient-based Ki image and the population-based Ki images

Among the population-based IF Ki images, there were significant differences in all three Ki parameters between Ki-350 and Ki-1000 images, between Ki-350 and Ki-700 images, and between Ki-700 and Ki-1000 images for both readers (all *p* < 0.001).

For both readers, significant strong and positive correlations (all *p* < 0.001) for Ki-max were noted between the reference individual patient-based IF Ki images and Ki-350 (reader 1, *r* = 0.93; reader 2, *r* = 0.90), Ki-700 (reader 1, *r* = 0.94; reader 2, *r* = 0.94), and Ki-1000 images (reader 1, *r* = 0.95; reader 2, *r* = 0.96) (Additional file 2: Fig. S2). Similarly, strong positive correlations were found for Ki-mean between the reference images and Ki-350 (reader 1, *r* = 0.91; reader 2; *r* = 0.89), Ki-700 (reader 1, *r* = 0.94; reader 2, *r* = 0.95) and Ki-1000 images (reader 1, *r* = 0.95; reader 2, *r* = 0.96) (Additional file 3: Fig. S3), and similar results were obtained for Ki-volume (Ki-350 images: reader 1, *r* = 0.84; reader 2, *r* = 0.75; Ki-700: reader 1, *r* = 0.82; reader 2, *r* = 0.73; Ki-1000: reader 1, *r* = 0.87; reader 2, *r* = 0.78) (Additional file 4: Fig. S4).

The Ki-1000 images displayed the largest *r*^2^ with the reference image for all Ki parameters for readers 1 and 2 (Ki-max, 0.91 and 0.92, respectively; Ki-mean, 0.91 and 0.92, respectively; and Ki-volume, 0.75 and 0.60, respectively).

### Inter-observer variability for Ki parameters

The inter-observer agreement for Ki parameters is summarized in Table 3. The inter-observer agreement was substantial to almost perfect for all Ki parameters between readers 1 and 2, ranging from 0.75 (95% CI = 0.64–0.85, Ki-volume of Ki-700 images) to 0.94 (95% CI = 0.91–0.97, Ki-max and Ki-mean of the reference images).

**Table 3:** *κ* values for inter-observer agreement for Ki parameters

	*κ* value^a^
*Individual patient-based Ki image*	
Ki-max	0.94 (95% CI = 0.91–0.97)
Ki-mean	0.94 (95% CI = 0.92–0.97)
Ki-volume	0.78 (95% CI = 0.67–0.89)
*Population-based Ki image*	
Ki-350 images	
Ki-max	0.81 (95% CI = 0.71–0.90)
Ki-mean	0.87 (95% CI = 0.80–0.94)
Ki-volume	0.76 (95% CI = 0.66–0.86)
Ki-700 images	
Ki-max	0.84 (95% CI = 0.77–0.91)
Ki-mean	0.92 (95% CI = 0.89–0.96)
Ki-volume	0.75 (95% CI = 0.64–0.85)
Ki-1000 images	
Ki-max	0.83 (95% CI = 0.75–0.91)
Ki-mean	0.90 (95% CI = 0.85–0.94)
Ki-volume	0.86 (95% CI = 0.80–0.92)

Ki-max, Maximum Ki; Ki-mean, Mean Ki; Ki-volume, Volume of Ki; and CI, Confidence interval

^a^Numbers in parentheses are 95% confidence intervals

## Discussion

This study evaluated the qualitative and quantitative findings of short-time-window (40–60 min) Ki images generated using the population-based IF and different BPL reconstruction methods using long-time-window (10–60 min) Ki images with the individual patient-based IF as the reference.

Individual patient-based IF Ki images with long-time dynamic scans have been considered difficult to generate in routine clinical practice because the injection must be performed with the patient in bed to measure the early phase of the IF [5, 6]. Conversely, population-based IF Ki images can be generated using a shortened time protocol, which obviates the need for the first dynamic scan for Patlak analysis [13, 14]. van Sluis et al. [13] examined whether the population-averaged IFs reduced the scan time to perform whole-body Patlak FDG PET imaging. They explored the effects of various simulated population-averaged IFs on the accuracy of Patlak analysis based on dynamic whole-body PET acquisition from 30 to 60 min. They reported that although there were percentages bias in Ki ranged from -16% to 16% using the simulated population-averaged IFs depending on the simulated amplitude and direction of the IF modifications, subsequent rescaling of the population-averaged IF reduced these Ki biases in most cases to < 5%. Thus, they concluded that scaling of a population-averaged IF to IF values seen in whole-body dynamic imaging from 30 to 60 min post-injection can provide accurate Ki estimates. Thus, population-based IF Ki imaging might represent a reasonable method with an easier protocol for Patlak analysis in routine clinical practice.

Meanwhile, the time window for Patlak analysis could have affected the qualitative and quantitative analysis [14] and Ki images with short-time scans might exhibit high noise levels [12]. Ye et al. [19] found that Ki derived from 0 to 90 min dynamic scans displayed larger area under the receiver operating characteristic curves than Ki derived from 0 to 60 min dynamic scans in lung nodule applications, and they indicated that the use of larger number of data time points could have added benefit when performing Patlak linear regression.

In our study, we adopted a long time window (10–60 min) for individual patient-based IF Ki images and a short time window (40–60 min) for population-based IF Ki images. Although the rating for positive and negative scans was identical between individual patient-based IF Ki images and all three types of population-based IF Ki images with a short time window, there were significant differences in the fraction of scans with “good conspicuity” (visual score 3) between individual patient-based IF Ki images and all three population-based IF Ki images, indicating that the image quality of population-based IF Ki images with a short time window was lower than that of individual patient-based IF Ki images with a long-time window.

BPL enables effective convergence of the image through an increased number of iterations by applying a penalization factor to suppress image noise, and this procedure was introduced to improve image quality through increasing the signal-to-noise ratio [15]. Image quality can be controlled by the penalization factor (beta value), which is the only user input variable in the algorithm. A high beta value increases the effect of regularization and strongly suppresses noise [20]. However, no study has examined whether the optimization of BPL reconstruction algorithms could improve the image quality in Ki images.

In our study, the number of “good conspicuity” scans was highest for Ki-1000 images and lowest for Ki-350 images, and there were significant differences in the number of “good conspicuity” scans between Ki-1000 and Ki-350 images. Based on linear regression analysis, the correlations of all Ki parameters between individual patient- and population-based IF Ki images were high. In addition, Ki-1000 images had the highest values of *r*^2^. These findings suggest that Ki-1000 images provide Ki values most similar to those of individual patient-based IF Ki image parameters. In addition to the substantial to almost perfect inter-observer agreement for all Ki parameters, population-based IF Ki images with a short time window reconstructed with a high beta value (1000 in this study) might represent an alternative to individual patient-based IF Ki images obtained with a long time window, even if the image quality of the population-based Ki-1000 images with a short time window was lower than that of individual patient-based Ki images with a long time window.

The fractional uptake ratio (FUR) has also been proposed as the quantitative simplified ^18^F-FDG metabolic rate estimation methods involving a single PET scan 45 min to 1 h post-injection and a complete input function [21].

It is an approximated value to Ki, and the FUR is calculated as a ratio of tissue activity *C*_*i*_ at time *T* and integral of plasma activity *C*_p_ from time 0 to *T* as the following formula [22, 23].

$${\text{FUR}} = C_{i} \left( {tk} \right)/\int\limits_{0}^{tk} {{\text{Cp}}\left( t \right){\text{d}}t}$$

On the other hand, as mentioned in the section of method, Ki is calculated as the following formula.

$${\text{Ki}} = \left( {C_{i} \left( {tk} \right){-}V_{{\text{p}}} C_{{\text{p}}} \left( {tk} \right)} \right)/\mathop \smallint \limits_{0}^{tk} C_{{\text{p}}} \left( t \right){\text{d}}t$$

Thus, the method of FUR assumes *V*_p_*C*_p_ (*tk*) is negligible with respect to *C*_i_ (*tk*). Knowing that at late times (45 to 60 min) *V*_p_*C*_p_ (*tk*) becomes negligible, because the concentration in the plasma is reduced at earlier time [23]. Thus, the FUR may be valid at late times but not at earlier times.

In our study, the population-based images were created by using the late times window (40 to 60 min). Thus, the population-based images might be replaced from Ki to FUR for the quantification of ^18^F-FDG metabolic rate. However, the relative accuracy of assuming negligible of *V*_p_*C*_p_ (*tk*) depending on the time [21] might challenge accurate quantification of ^18^F-FDG metabolic rate. Moreover, generally, the blood sampling from the injection to the scan is required for calculating the FUR [22]. In our study, we applied the Ki for the quantification of ^18^F-FDG metabolic rate, and it might be less time dependence compared with FUR.

This study had some limitations that should be considered when interpreting our results. First, this was a retrospective study with a relatively small sample size. Therefore, a prospective study with a larger sample is needed to confirm the validity of the present findings. Second, the four different types of Ki images were read simultaneously for each study, which may have led to a biased qualitative assessment. Third, motion artifact correction was not performed because we could not develop the optical tracking technique to correct for motion in list-mode PET. The accuracy of IDIFs is affected by body motion and partial volume effects [14, 24, 25]. Thus, the influence of motion effect could not be ignored in the quantitative analysis. However, to create the precious VOI for the quantitative analyses, the registration of CT and reconstructed dynamic PET image was verified using ACQC software on the PET/CT scanner. We confirmed that there was no incorrect registration of CT and reconstructed dynamic PET image. Thus, we considered that the effects of body motion might be less in the results of quantitative analyses. Indeed, all Ki parameters exhibited the substantial to almost perfect inter-observer agreements. Fourth, there is no consensus regarding the number of individual patient IF samples needed to generate the population-based IF. In this study, we generated the population-based IF using the regression equation acquired from the correlations between IDIF $\left( {\mathop \smallint \limits_{0}^{50} C_{{\text{p}}} \left( t \right){\text{d}}t} \right)$ and Ao-50 among the 12 patients. Indeed, there were quite differences in all 3 Ki values (Ki-max. Ki-mean and Ki-volume) between individual patient- and population-based IF Ki images on both readers. It has been reported that the time window for the Patlak analysis could have affected the quantitative analysis [14], and as mentioned former, Ki images with short-time scans might exhibit high noise levels [12]. Thus, not only applying different time windows but also applying different IFs might influence the differences in Ki values between individual patient-based Ki and population-based Ki values. However, the correlations of all parameters between the reference Ki images (individual patient-based IF Ki images) and three types of population-based IF Ki images were high for both readers. The generation of the population-based IF from IF data of a small number of patients (16 patients) has also been reported [19]. Moreover, in the 12-patient study, the influence of the interaction between individual patient- and population-based IF Ki images might not be ignored. Thus, further research is required to clarify the number of individual patient IF data points which are sufficient to generate the population-based IF. Fifth, the follow-up Ki images using the previously acquired IDIF were not created which might have also the potential to reduce the scan time for Patlak analysis. In this study, the feasibility of short-time-window Ki images using a population-based IF was only examined. Thus, further research is required whether the adaption of previously acquired IDIF can reduce the scan time for creating the follow-up Ki images. Sixth, only three beta values (350, 700, and 1000) were used to optimize the BPL algorithm. The use of higher beta values might yield different results, and further studies seeking to optimize reconstruction protocols for Ki imaging are warranted. Finally, we did not examine the relationship between clinical findings such as the disease activity of CS and the Ki images. Clinical assessment of CS was beyond the scope of this feasibility study. Thus, further study is needed to clarify whether the short-time-window population-based Ki images are useful for the clinical assessment of patients with CS.

## Conclusions

Our study revealed that short-time-window Ki images with a population-based IF reconstructed using the BPL algorithm with a high beta value (1000 in this study) were closely correlated with long-time-window Ki images generated using an individual patient-based IF. Short-time-window Ki images using a population-based IF and BPL reconstruction might represent a practical alternative to long-time-window Ki images generated using an individual patient-based IF.

## Supplementary Information

**Additional file 1. Figure S1.** The correlation between IDIF and Ao-50 among the 12 patients. There is a significantly high correlation between IDIF and Ao-50 (*Y *= 113.76 + 97.16*x*; *r *= 0.98, *p *< 0.001).

**Additional file 2. Figure S2.** Correlations of the Ki-max between the reference images and the population-based IF Ki images. For both readers, significant strong and positive correlations (all *p* < 0.001) for Ki-max were noted between the reference images and Ki-350 (**a** reader 1, *r* = 0.93; **b** reader 2, *r* = 0.90), Ki-700 (**c** reader 1, *r* = 0.94; **d** reader 2, r = 0.94), and Ki-1000 images (**e** reader 1, *r* = 0.95; **f** reader 2, *r* = 0.96).

**Additional file 3. Figure S3.** Correlations of the Ki-mean between the reference images and the population-based Ki IF images. For both readers, significant strong and positive correlations (all *p* < 0.001) for Ki-mean were noted between the reference images and Ki-350 (**a** reader 1, *r* = 0.91; **b** reader 2; *r* = 0.89), Ki-700 (**c** reader 1, *r* = 0.94; **d** reader 2, *r* = 0.95) and Ki-1000 images (**e** reader 1, *r* = 0.95; **f** reader 2, *r* = 0.96).

**Additional file 4. Figure S4.** Correlations of the Ki-volume between the reference images and population-based Ki images. For both readers, significant and positive correlations (all *p* < 0.001) for Ki-volume were noted between the reference images and Ki-350 (**a** reader 1, *r* = 0.84; **b** reader 2, *r* = 0.75), Ki-700 (**c** reader 1, *r* = 0.82; **d** reader 2, *r* = 0.73) and Ki-1000 images (**e** reader 1, *r* = 0.87; **f** reader 2, *r* = 0.78).

## Data Availability

The datasets used and/or analyzed during the current study are available from the corresponding author on reasonable request.

## Abbreviations

BPL: Bayesian penalized likelihood
CS: Cardiac sarcoidosis
CT: Computed tomography
^18^F-FDG: ^18^F-fluorodeoxyglucose
FOV: Field of view
IDIF: Image-derived input function
IF: Input function
Ki-max: Maximum Ki
Ki-mean: Mean Ki
Ki-volume: Volume of Ki
PET: Positron emission tomography
ROC: Receiver operating characteristic
ROI: Region of interest
SD: Standard deviation
SUV: Standardized uptake value
VOI: Volume of interest
