# Supplementary figures and images for: Short-time-window Patlak imaging using a population-based arterial input function and optimized Bayesian penalized likelihood reconstruction: a feasibility study
Source: EJNMMI Res. 2022 Sep 8;12:57. doi: 10.1186/s13550-022-00933-8 (PMC9458796; doi:10.1186/s13550-022-00933-8)

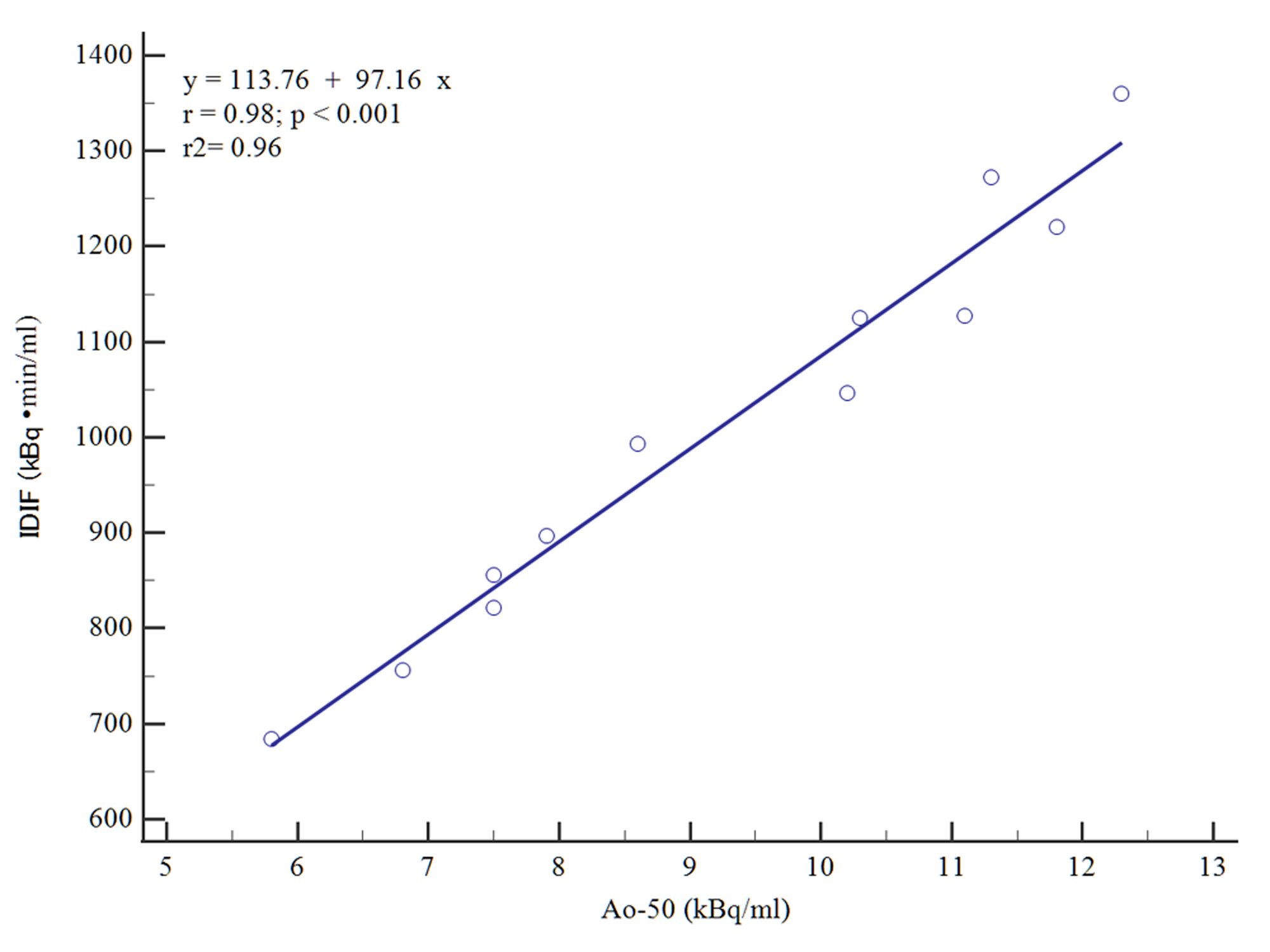

Supplement: Supplementary file 1 — Additional file 1. Figure S1. The correlation between IDIF and Ao-50 among the 12 patients. There is a significantly high correlation between IDIF and Ao-50 (Y = 113.76 + 97.16x; r = 0.98, p < 0.001). [file 13550_2022_933_MOESM1_ESM.tif]

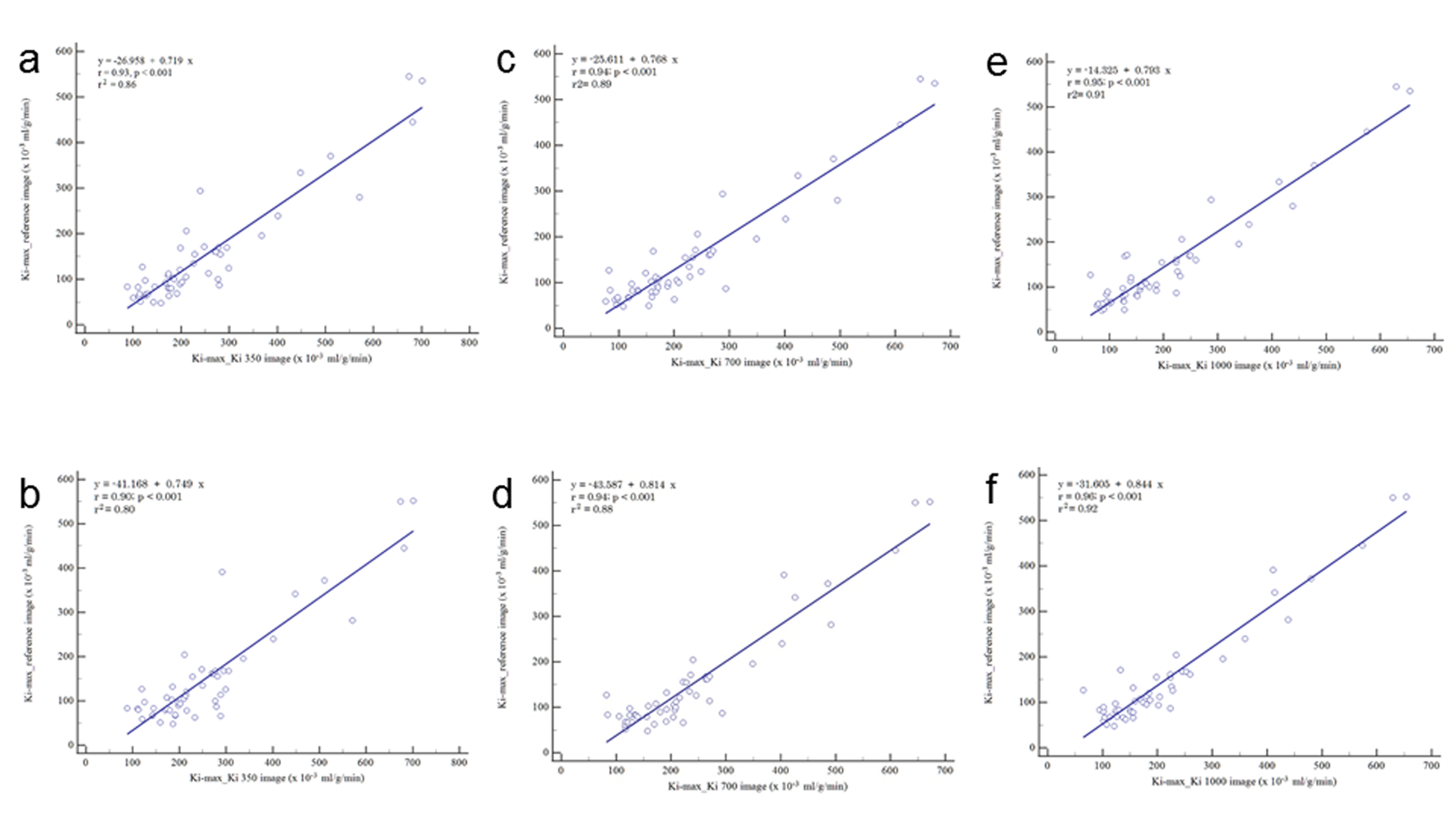

Supplement: Supplementary file 2 — Additional file 2. Figure S2. Correlations of the Ki-max between the reference images and the population-based IF Ki images. For both readers, significant strong and positive correlations (all p < 0.001) for Ki-max were noted between the reference images and Ki-350 (a reader 1, r = 0.93; b reader 2, r = 0.90), Ki-700 (c reader 1, r = 0.94; d reader 2, r = 0.94), and Ki-1000 images (e reader 1, r = 0.95; f reader 2, r = 0.96). [file 13550_2022_933_MOESM2_ESM.tif]

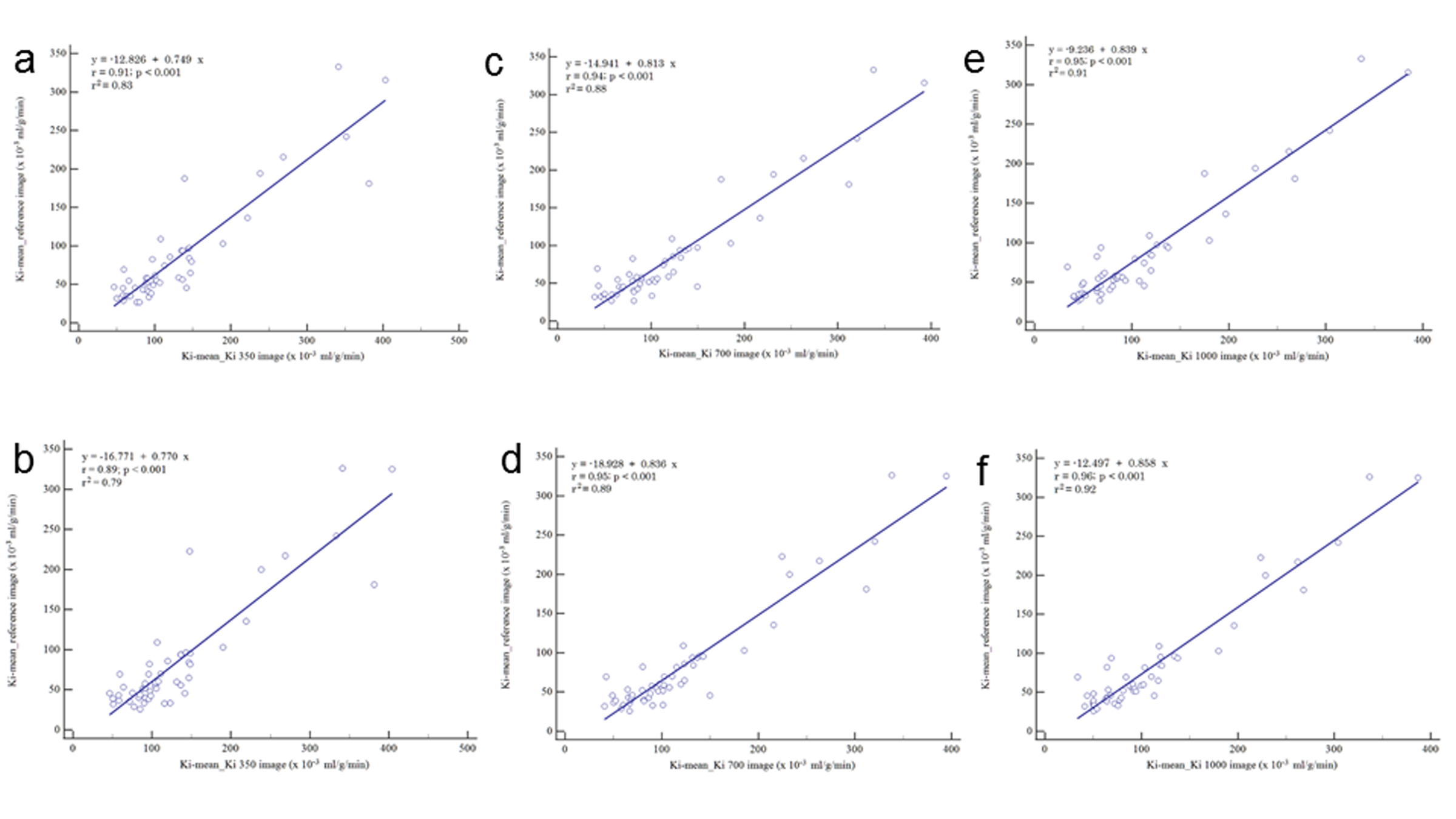

Supplement: Supplementary file 3 — Additional file 3. Figure S3. Correlations of the Ki-mean between the reference images and the population-based Ki IF images. For both readers, significant strong and positive correlations (all p < 0.001) for Ki-mean were noted between the reference images and Ki-350 (a reader 1, r = 0.91; b reader 2; r = 0.89), Ki-700 (c reader 1, r = 0.94; d reader 2, r = 0.95) and Ki-1000 images (e reader 1, r = 0.95; f reader 2, r = 0.96). [file 13550_2022_933_MOESM3_ESM.tif]

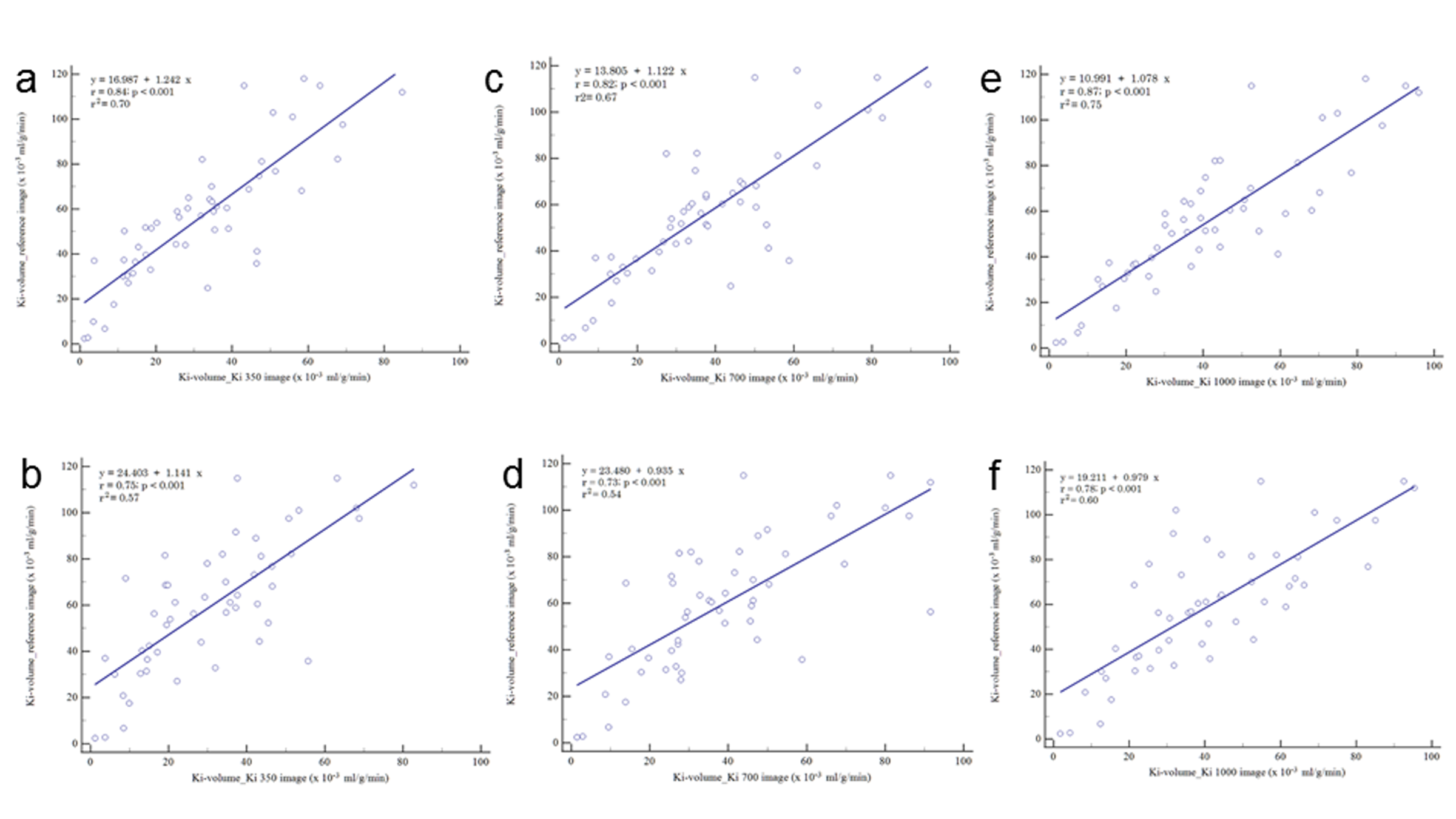

Supplement: Supplementary file 4 — Additional file 4. Figure S4. Correlations of the Ki-volume between the reference images and population-based Ki images. For both readers, significant and positive correlations (all p < 0.001) for Ki-volume were noted between the reference images and Ki-350 (a reader 1, r = 0.84; b reader 2, r = 0.75), Ki-700 (c reader 1, r = 0.82; d reader 2, r = 0.73) and Ki-1000 images (e reader 1, r = 0.87; f reader 2, r = 0.78). [file 13550_2022_933_MOESM4_ESM.tif]
